# Sclereids are strong enough to support the delicate corollas: experimental and computational data evidence from *Camellia sinensis* (L.)

**DOI:** 10.1038/srep43788

**Published:** 2017-03-02

**Authors:** Wei Zhang, Yuanyuan Xue, Shuo Yang, Yangang Wang, Hong Zhao

**Affiliations:** 1 Marine College, Shandong University at Weihai, Weihai 264209, China; 2 State Key Laboratory of Dao-di Herbs, National Resource Center for Chinese Materia Medica, China Academy of Chinese Medical Sciences, Beijing 100700, China; 3 Research Center of Mechanics and Mechatronic Equipment, Shandong University at Weihai, Weihai 264209, China

## Abstract

Sclereids are a fundamental cell type that widely exist in higher plants and are generally thought to have a mechanical function. However, the occurrence of sclereids in the ephemeral corolla has rarely been documented and their biological significance is poorly understood. In this study, flower buds from *Camellia sinensis* at various ontogenetic stages were sampled, cleared, sectioned, stained, and examined using light microscopy to ascertain the morphology and distribution of sclereids and their variation. In addition, *Camellia japonica* plants with distinctive floral structures were investigated and compared to explore whether sclereid occurrence is associated with floral form. In particular, a computational simulation using finite element analysis was undertaken to investigate how corollas, with and without sclereids, responded to wind and rain. The results showed that sclereids have some mechanical properties that are based on their shape and distribution, which make the soft corolla strong enough to protect the inner ovary. Thus, corolla sclereids may explain how the seemingly delicate corolla performs its protective function in response to environmental stresses. These findings provide further evidence for the hypothesis that flower traits exhibit adaptive responses to abiotic factors in addition to their traditionally recognized pollinator-mediated selection.

The corolla is the showiest part of plant and has been traditionally recognized as an organ for attracting pollinators because most of its positive attributes of color, shape, size, and scent are associated with this^1^. However, new evidence suggests that flowers may adapt to more pluralistic factors, including not only pollinators and herbivores, but also their abiotic environment^2^,^3^,^4^. The corolla may have protective as well as attraction functions because the reproductive organs are always sheltered under the enclosed corolla until they are fully mature.

Rain and wind are two of the most common environmental stresses, and can strongly affect plant development, growth, and reproduction^5^,^6^. Many plants have evolved various morphological traits to protect against the deleterious effects of rain and wind. For example, vegetative organs can reduce elongation growth, and promote thickening growth and root resource allocation to reduce the effects of wind^7^. Downward-facing flowers have evolved their bracts or corollas to function as shielding umbrellas^8^, while some upright flowers close their corolla to shelter the stamens from rain, which may dilute floral nectar, remove pollen, and decrease plant viability^4^,^9^.

Traditionally, it has been generally thought that the corolla is simply composed of several layers of parenchyma cells and lacked any special mechanical tissue^10^, although vascular bundles in the petals that contain lignified vessels and sclerenchymatous fibers may create a supporting scaffold for parenchyma tissue. Therefore, there are relatively little data to explain how the delicate corolla acts as a protective organ against rain and wind. Recently, the occurrence of sclereids has been reported in some petals of *Camellia* species and this may partially explain the mechanical properties of the corolla^11^. This study was carried out on the mature petal, when the flower had fully expanded, and its pollen had matured. However, it is unclear how the corolla performs its protective function in the early stages of flower development when all the petals are closed and act as a rain shelter. The petal primordia (the petals at the pre-anthesis stage) at this point have a vital protective function if the species do not produce sepals, but little is known about their anatomical structure, especially the sclereid architecture in the immature petals.

Finite element analysis (FEA) is a numerical method for solving problems that are characterized by partial differential equations. It has become one of the most powerful tools in mechanical engineering disciplines because this method can be applied to problems of great complexity and unusual geometry, it allows detailed visualization of where structures bend or twist, and it shows the distribution of stresses and displacements^12^. Furthermore, the availability of fast computers allows problems that are intractable using analytical or mechanical methods to be solved in a straightforward manner using FEA. In this study, we explored the mechanical properties of the *Camellia* corolla using both experimental and FEA methods. We focused on the spatial architecture of sclereids in the unopened corolla in order to understand the mechanism underlying the corolla protective function that is enhanced by sclereid occurrence. The multidiscipline research methods used in this study could provide unique insights into how the flower responds to external pressures and thus may provide further evidence for the plant functional-structural hypothesis.

## Results

### Sclereids in different parts of the *Camellia sinensis* flower bud and at different developmental stages

**Figure 1:**
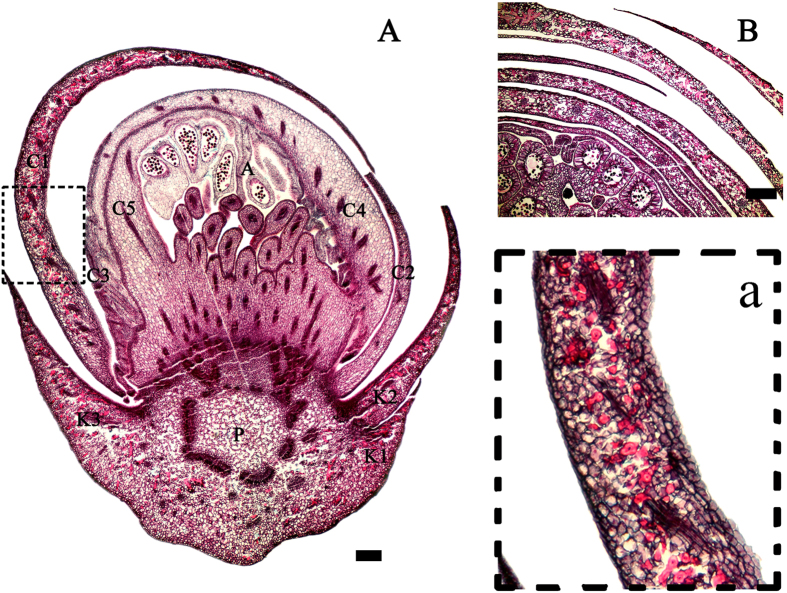
Sclereids and their distribution in different parts of *Camellia sinensis* flower buds. (**A**) longitudinal section of a whole bud at about 20 days before flowering, P = pedicel, K = calyx, C = corolla, A = Androecium; (**B**) transverse section of the median region of a floral bud at about 20 days before flowering. The petals have folded and partly overlap, which means there are at least four section layers that contain sclereids. a: enlarged section of A showing the distribution and the incomplete shape of the sclereids. Most sclereids show a cross-section of their branches. This is in contrast to Fig. 3D. Scale bars = 400 μm.

**Figure 2:**
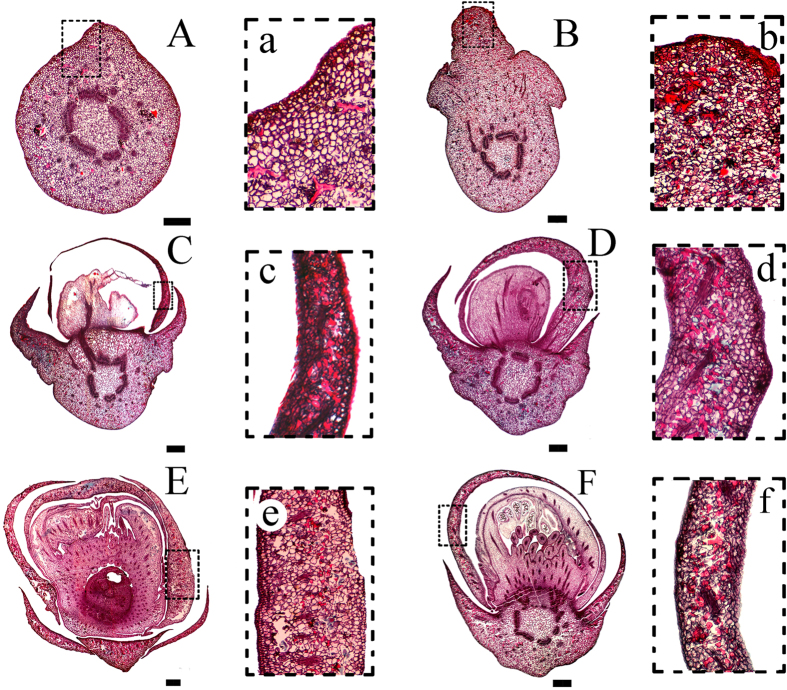
Distribution of sclereids at the different flower bud developmental stages in *C. sinensis*. (**A**–**F**) longitudinal sections of buds from younger to older growth stages, (**A**) the floral primordium stage, (**B**) the sepal stage, (**C**,**D**) the first petal primordium stage, (**E**,**F**) the second petal primordium stage; a–f: correspondingly enlarged sections of (**A**–**F**) showing some sclereid features. Scale bars = 800 μm.

**Figure 3:**
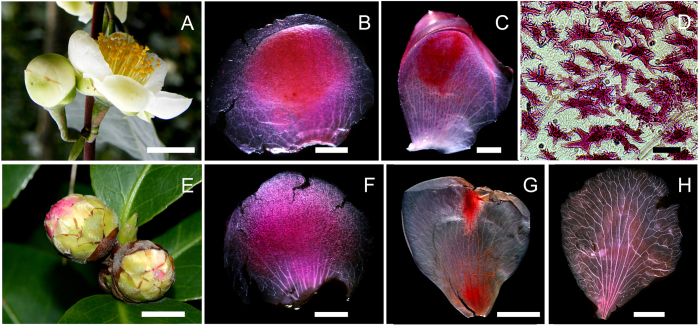
Distribution of sclereids in the two *Camellia* species. (**A**–**D**) *C. sinensis*, (**A**) flower and bud, (**B**) sepal, and (**C**) petal primordium; (**D**) amplification of (**C**), also shows the sclereid forms and types seen in (**B**,**F**), and (**G**); (**E**–**H**) *C. japonica*, (**E**) buds, (**F**) sepal, (**G** and **H**) petal primordium. Scale bars: (**A**,**E**,**G**) = 1 cm; (**B**) = 1 mm; (**C**,**F**) = 2 mm; (**D**) = 100 μm; (**H**) = 5 mm.

Sclereids were easily observed in the parenchyma tissue of the flower bud section because of their large size, unique shapes, and intense coloration, which sharply demarcated them from their neighboring cells. In contrast to the calyx, they were mainly located in the 1–2 layers of the outer petal primordia, according to the transverse, longitudinal, and tangential sections (Fig. 1, Fig. S1). Furthermore, serial sections from different developmental stages of the flower bud showed that sclereid formation can be traced back to the floral primordium stage. They first differentiated in the pedicel, and then sequentially associated with the differentiation procedure for flower organs, which is primordium development, followed by sepals, the first outer petal primordia, etc. (Fig. 2). It seemed that the sclereids always filled the outer layers of the flower bud during floral development to ensure they covered the area above the stamen and pistil. In these floral organs, sclereids were densely located in the mesophyll tissue, where their large, main bodies penetrated through the intercellular spaces between the parenchyma. The sclereids in this area were mainly polymorphic and possessed a column base with branches at either or both ends. The branches of the sclereids were usually entangled and they often extended into the intercellular spaces. They functioned as skeleton structures, uniting the adjacent cells and tissues (Figs 1A and 3D).

### Sclereids in the cleared flower bud laminae of the two species

Sclereids were conspicuous in the cleared petal primordia and sepals because they were often aggregated and showed intense red coloration after they had been stained with safranin (Fig. 3). The large size (ca. 100–300 μm) of the isolated sclereids meant that they were even visible to the naked eye as small, red points. The intact sclereid shapes were clearly observed in the transparent petal primordium blade by microscopy. They mainly consisted of polymorphic forms (Fig. 3D). Both the sepals of *C. sinensis* and *C. japonica* contained a large number of sclereids (Fig. 3B,F). However, the petal surface of the *C. sinensis* primordia, a species with reduced leathery sepals, was made up of areas that contained at least 50% sclereid-tissue (Fig. 3A,C), whereas it represented only up to 5% (0% in most cases) of the *C. japonica*, petal surface area, a species with robust, crustaceous sepals (Fig. 3E,G,H). Furthermore, the sclereids were present in smaller quantities on the edges and over the whole petal area, but were concentrated in the central part of the sepals or closer to the top of the petal primordia (Fig. 3B,C,F,G). When the sepals and petal primordia were closed during the flower bud stage, these regions overlapped and formed a rigid shell, which gave additional reinforcement against physical forces that may damage the reproductive organ.

### Computational simulation of sclereid mechanical properties

**Figure 4:**
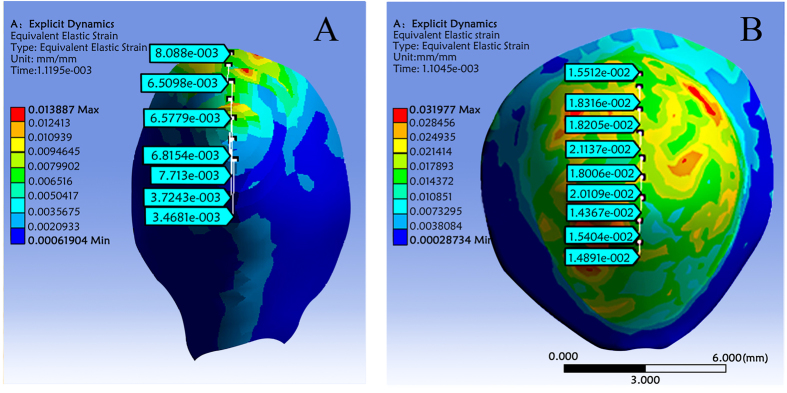
Equivalent elastic strain values and their distributions derived from using different finite element models in Ansys workbench. (**A**) Simulation of *C. sinensis* corollas with sclereids; (**B**) Simulation *C. japonica* corollas without sclereids. Color gradients represent equivalent elastic strain levels from high (red) to low (blue).

We constructed two finite element models (FEM) that simulated the loading scenarios for *C. sinensis* corollas with sclereids (FEM-A) and *C. japonica* corollas without sclereids (FEM-B). A total of 114,984/70,308 elements and 23,691/22,871 nodes were generated in each model, respectively. In addition, the model statistics showed that their meshing qualities were 0.8047 ± 0.1381/0.7859 ± 0.1483 and their skewness were 0.2691 ± 0.1762/0.2962 ± 0.1862, respectively, which indicated that the meshing was adequate and of a high-quality (Table S1). Each model was used to explore the corolla response to the applied rain force by measuring the equivalent elastic strain values and their distributions. Our results showed that the two models differed greatly for these parameters. In FEM-A, the largest deformation occurred mainly in the apical part of model and decreased gradually from the center to the edges (Fig. 4A). This strain distribution pattern correlated well with the corolla sclereid distribution (Fig. 4A vs. Fig. 3C). In contrast, the largest deformation was generally scattered in FEM-B and had no such regularity (Fig. 4B). In addition, the results showed that FEM-B deformation was greater than FEM-A under the same applied force. For example, the maximum strain value in FEM-B was 2.3-fold higher than that in FEM-A (0.031977/0.013887, respectively). Therefore, the FEA results showed that the maximum strain locations predicted by the finite element model correlated well with the corolla sclereid distribution, and corollas that contained sclereids were strong enough to withstand these maximum strains because they suffered less deformation compared to corollas without sclereids.

## Discussion

Sclereids are widespread among a broad range of higher plants^13^. They are widely distributed throughout the plant body, including the stems, leaves, fruits, and seeds, and are generally thought to have a mechanical function because of their strongly lignified and thick-walled forms^10^. However, the occurrence of sclereids in the petals was very low. Until recently, they have only been reported in a relatively small number of plants, such as Melastomaceae^14^, Nymphaeaceae^15^, Loranthaceae^16^, and Loganiaceae^17^. Therefore, experimental studies on the specific, adaptive advantages of sclereids are scarce, especially with regards to their occurrence in the ephemeral corolla. Previous studies have shown that *Camellia* plants are rich in sclereids, and their leaves, petioles, sepals, and petals all have sclereids^11^,^18^,^19^,^20^,^21^,^22^,^23^. In this study, *C. sinensis* lacked stout calyxes, which meant that the growing flower bud becomes increasingly exposed to external stresses, such as rain and wind, which can be fatal. Fortunately, the ovaries at this stage are always under the protection of the folded and curling corolla until they are mature. Our anatomical data from serial sections of different development stages showed that sclereids differentiated early from the parenchyma tissue and were associated with the differentiation procedure for flower organs. This meant that they were present in the reproductive organs from the start of organ development until the end of reproduction. In addition, their shape and unique distribution pattern led to improved mechanical properties, which meant they could function as ‘accessory’ cells that stiffened the delicate corolla. Correspondingly, an alternative virtual approach using FEA was carried out to test the merits of mechanical traits related to sclereids. The FEA method originated from engineering principles, but has recently been introduced and employed to investigate the biomechanical properties of plants. This method has been used most frequently to determine the force-deformation behavior of grain, fruit, and vegetable tissue under external loading to improve postharvest handling practices. Some examples include sunflower grains^24^, tomato^25^, apple^26^, and wheat kernels^27^. In addition, a number of studies on tree shapes and growth stresses have also used the FEA method to investigate tree mechanical behavior under internal or wind-induced pressures^28^,^29^,^30^. Hamm *et al*. applied the FEA method to quantitatively explore the diatom shell form-function relationships and provided a promising numerical way to elucidate why diatoms have evolved their shape and structure in the way that they have^31^. However, studies that have modeled the mechanical behavior of flowers are scarce. To our best knowledge, this paper is the first study that has undertaken a biomechanical analysis of corollas using the FEA method. The simulation results showed that sclereids considerably strengthened the corolla, which may enable it to withstand more external stress to its thin walled, softer cells. Further research in this field is required. However, these results provide encouraging evidence that might be difficult or impossible to detect using routine methods, such as morphological and anatomical methods.

An increasing number of studies have suggested that flower form and size is a selective process that includes the physical environment as well as biotic pollinators and herbivores^4^,^32^. Entomophilous flowers have to perform two seemingly antithetic tasks to enhance reproductive success: pollinator attraction and ovary protection^33^. Traditionally, it was considered that these partly opposing functions were assigned to different flower parts. The sepals had a protection function and the petals were an attractant^34^. However, our study revealed that when reinforced by sclereids, the corolla can act as a protective organ in addition to its usual pollinator attraction role when the calyx is small in size. Structural reinforcement can be costly and is achieved at the expense of metabolic investment, making the trade-off between support and metabolic supply a complex process. Indeed, *Camellia* plants have evolved their flower structures in a very sophisticated, but compromising way. *C. japonica*, which have no conspicuous corolla sclereids, have developed a short, stout pedicle and a number of robust crustaceous sepals, and the sepals and petals differ only slightly in size and shape (Fig. 3E). In contrast, *C. sinensis*, which has conspicuous corolla sclereids, has developed a long pedicle and small number of reduced leathery sepals (Fig. 3A). Therefore these results illustrate the morphological and functional diversities of the corolla, which may be shaped by their physical environment as well as the needs of their pollinators.

## Materials and Methods

### Taxa sampling

The genus *Camellia* contains more than 120 species and can be subdivided into two groups based on the pedicle length and the degree of differentiation between sepals and petals^35^. In this study, we sampled a representative of both groups. *Camellia sinensis* (L.) Kuntze has a long pedicle and reduced, leathery sepals, with sepals and petals distinct from each other. In contrast, *Camellia japonica* (L.) has a short, stout pedicle and robust, crustaceous sepals, with sepals and petals only slightly distinct from each other (with a gradual progression in size and shape). Flower buds from the two species, at different developmental stages, i.e. the floral primordium stage, the sepal stage (the first whorl has appeared), the first petal stage (the second whorl has appeared) and the second petal stage (the third whorl has appeared), were collected from Weihai in northern China (37°20′30.89″N, E122°10′44.78E) (temperate monsoon climate, average temperature 11.8 °C, and average humidity 60% during flowering) and Jinggangshan in southern China (26°35′49.454″N, 114°08′22.432″E) (sub tropical monsoon climate, average temperature 14.5 °C, and average humidity 80% during flowering). The fresh materials were fixed in formalin (37–40%)–acetic acid–alcohol (50%) solution (5:5:90, v/v/v) (FAA) for at least two days and stored in 70% ethanol.

### Tissue clearing and sectioning

Clearing removes all the pigments and allows staining with safranin or substitutes to show up the sclereids in the petal blade. In this study, we cleared samples using a modified method from Ellis *et al*.^36^. The petals or sepals stored in FAA were rinsed in distilled water, immersed in 2% NaOH and left overnight in a 40 °C oven. The NaOH solution was changed twice a day until the petals lost all their pigments and looked clear. The process may take 3−5 days, depending on the thickness and texture of the materials. After this process, the petals became soft and fragile and were carefully transferred into 15% NaClO for 1 h to remove any cloudiness. The cleared petals were rinsed in distilled water, dehydrated through an ethanol series (50, 70, 95, 100, 100%; 1 h each step), stained with 1% safranin (4 h) and 0.5% fast green (1 min), and then they were photographed. Paraffin sectioning was used to examine the sclereids in the perianth tissue. Five typical buds at each developmental stage were selected and hand-cut longitudinally into two equal sections. These sections were dehydrated through an ethanol series (50, 70, 95, 100, 100%; 1 h each step), then transferred to a xylene-ethanol series (3:1, 1:1, 1:3, 0:1, v/v; 1 h each step), and finally saturated with paraffin. The materials embedded in the paraffin wax were sectioned with a rotary microtome (Leica RM2235, Leica Microsystems, Heidelberg, Germany). Transverse, longitudinal, and tangential serial sections were cut at a thickness of 20 μm and stained with safranin for 4 h and fast green for 1 min. Then they were mounted on slides with neutral balsam for observation. The serial sections were examined using a light microscope (Axio imagerA1, Carl Zeiss Co., Shanghai, China).

### Finite element modelling and calculations

A finite element analysis was performed in order to better visualize and understand the mechanical properties of sclereids in the corolla. In this analysis, the average size of the corolla was determined by randomly picking 100 *C. sinensis* petals and then measuring their three major dimensions, namely length, width, and thickness, using a digital caliper. A geometric model of the corolla was created by computer-aided design (CAD) software (Solidworks, Dassault Systemes, Solidworks Corps, Concord, MA, USA). The model was then imported into Ansys Workbench 15.0 (Ansys Inc., Canonsburg, PA, USA) for finite element simulation. The finite element model of the corolla structure was divided into shell63 and beam4 elements, which separately simulated parenchymatous tissue and sclereids. When meshing the elements, the relevance was set to 0, the relevance center was set to ‘coarse’. The corolla material was considered to be homogeneous, the Yong’s modulus (*E*) of the sclereids was set to 0.5 GPa, and the Poisson’s ratio (v) was set to 0.4, which was comparable with the fibers (*E* = 0.49–1.0, v = 0.5). In addition, a finite element model for raindrops was also established according to previous studies^37^,^38^,^39^, which set their shapes as an approximate sphere with a diameter of 2 mm and a terminal velocity of 6 m/s (Fig. S2). Finally, mechanical response analysis of the corolla was carried out using the finite element model under raindrop loading.

## Additional Information

**How to cite this article**: Zhang, W. *et al*. Sclereids are strong enough to support the delicate corollas: experimental and computational data evidence from *Camellia sinensis* (L.). *Sci. Rep.* **7**, 43788; doi: 10.1038/srep43788 (2017).

**Publisher's note:** Springer Nature remains neutral with regard to jurisdictional claims in published maps and institutional affiliations.

## Supplementary Material

Supplementary Information
