# Supplementary material for: Sclereids are strong enough to support the delicate corollas: experimental and computational data evidence from Camellia sinensis (L.)
Source: Sci Rep. 2017 Mar 2;7:43788. doi: 10.1038/srep43788 (PMC5333139; doi:10.1038/srep43788)
Supplement: Supplementary Information [file srep43788-s1.pdf]

Sclereids are strong enough to support the delicate corollas: experimental and computational data evidence from *Camellia sinensis* (L.)

Wei Zhang[1,2], Yuanyuan Xue[1], Shuo Yang[1] Yangang Wang[3] & Hong Zhao[1]

Supplementary Table S1. Statistical analyses of meshing for the two finite element models

		**FEM-A model**	**FEM-B model**
	Elements	114984	70308
Mesh size	Nodes	23691	22871
	Min	0.0850	0.1101
	Max	0.9999	0.9992
	Average	0.8047	0.7859
Element quality	Standard deviation	0.1381	0.1483
	Min	0.0972	0.0015
	Max	0.9965	0.9992
	Average	0.2691	0.2962
Skewness	Standard deviation	0.1762	0.1862

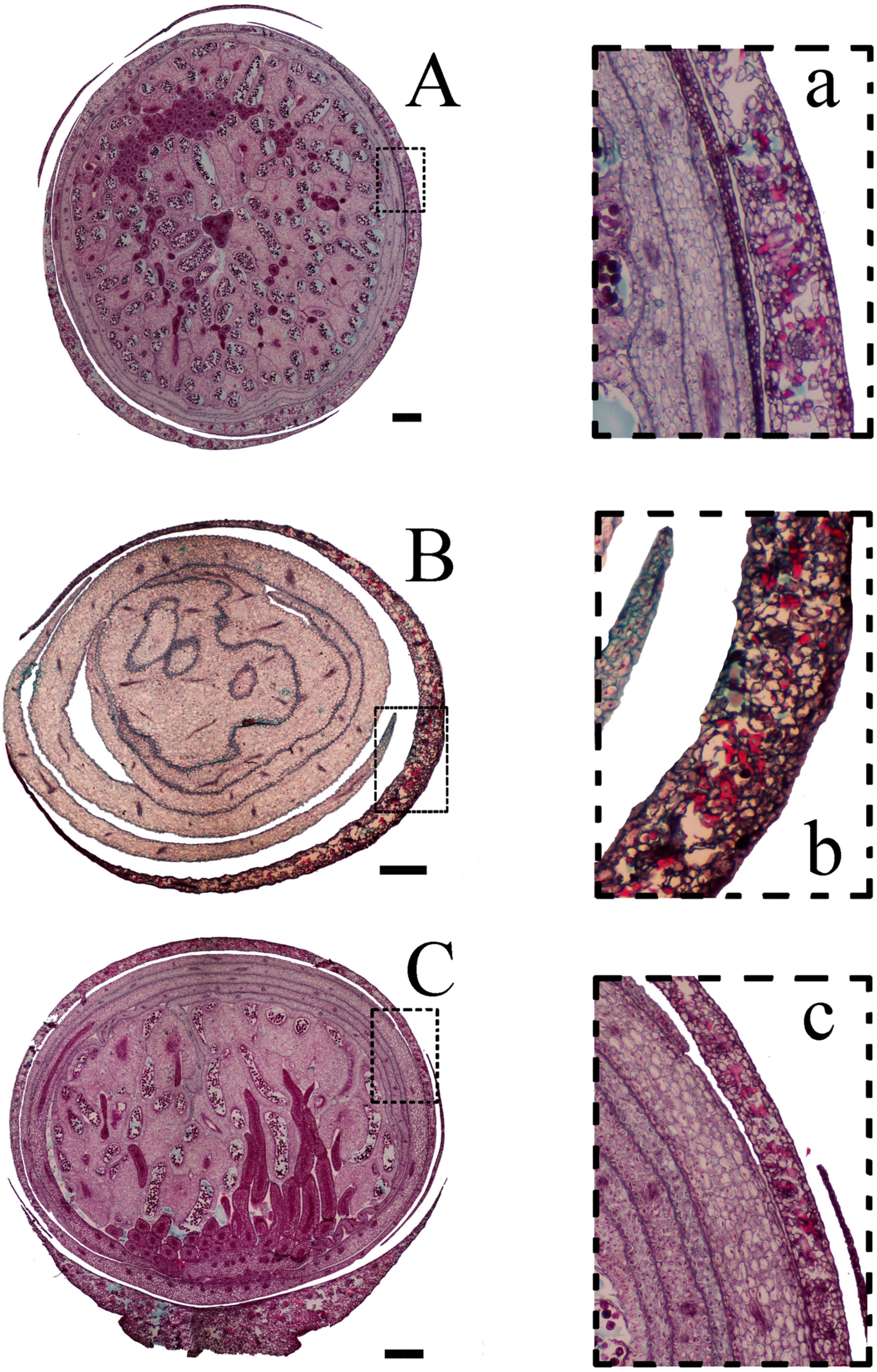

Supplementary figure S1. Whole paraffin section mounts showing that the sclereids distributed in the outer layer of the corolla. A: transverse section from the median region of the floral bud, B: tangential section at the apex of the bud, C: longitudinal section along the longitudinal axis of the bud; a, b, and c are correspondingly enlarged sections of A, B, and C showing some sclereid features. All sections were taken from C. sinensis at the floral stage about 20 days before flowering. Scale bars = 800 μm.

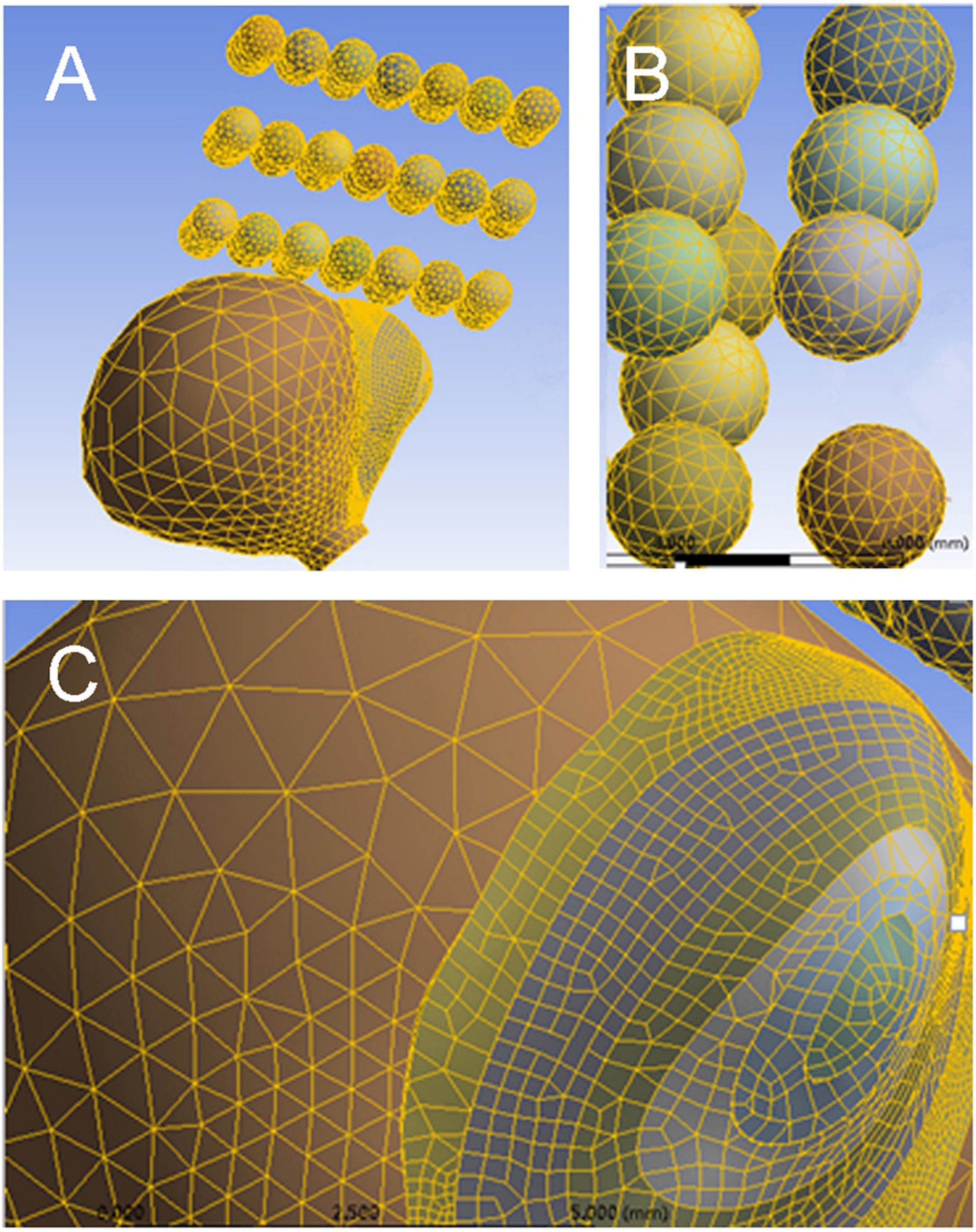

Supplementary figure S2. A meshed model simulating the corolla under raindrop loading. A: The whole figure shows the loading mode for rain with wind. B: enlarged section of the raindrops showing their shapes and finite element meshing mode. C: enlarged section of the corolla showing the finite element meshing mode. All images were generated from Ansys Workbench.
